# Smoking is an independent risk factor for stricture recurrence after the urethroplasty: a systematic review and meta-analysis

**DOI:** 10.1590/S1677-5538.IBJU.2022.0244

**Published:** 2022-08-20

**Authors:** Yu-cheng Ma, Lede Lin, Zhumei Luo, Tao Jin

**Affiliations:** 1 Sichuan University West China Hospital Institute of Urology Chengdu Sichuan China Department of Urology, Institute of Urology (Laboratory of Reconstructive Urology), West China Hospital, Sichuan University, Chengdu, Sichuan, China;; 2 Third People's Hospital of Chengdu Department of Oncology Sichuan China Department of Oncology, the Third People's Hospital of Chengdu, Sichuan, China

**Keywords:** Urethral stricture, urethroplasty, risk factor, smoking, meta-analysis

## Abstract

**Objective:**

To clarify the association between smoking and stricture recurrence after urethroplasty.

**Materials and Methods:**

Pubmed, Web of Science, Embase, and Cochrane databases were searched with keywords: “urethroplasty,” “buccal mucosa graft urethroplasty,” “oral mucosa graft urethroplasty,” “excision and primary anastomosis urethroplasty,” “urethral stricture recurrence” until July 1, 2022. Inclusion and exclusion criteria were based on PICOS principles. The quality of included studies was assessed by Newcastle-Ottawa Scale (N.O.S.) system. Hazard ratio (H.R.), odds ratio (OR), and relative risk (RR) with 95% confidence interval (CI) were extracted or re-calculated from included studies. Meta-analysis was performed with Stata 15.0 based on univariate and multivariate data separately. Sensitivity analysis was performed to test the stability of the meta-analysis. I2 was calculated to evaluate heterogeneity. Publication biases were assessed by Egger's and Begg's tests. Funnel plots of univariate analysis and multivariate analysis were also offered.

**Results:**

Twenty one studies with 6791 patients were involved in this meta-analysis. The analysis results of the two stages were consistent. In the univariate meta-analysis stage, 18 studies with 5811 patients were pooled, and the result indicated that smoking might promote stricture recurrence (RR=1.32, P=0.001). Based on the adjusted estimate, 11 studies with 3176 patients were pooled in the multivariate meta-analysis stage, and the result indicated that smoking might promote stricture recurrence (RR=1.35, P=0.049). There was no significant heterogeneity in both the univariate and multivariate stages.

**Conclusion:**

Our study demonstrates that smoking may prompt stricture recurrence after the urethroplasty. Quitting smoking may be a good option for patients undergoing urethroplasty surgery.

## INTRODUCTION

When urologists deal with the urethral stricture patients by urethroplasty, one of the most worrying situations is the stricture recurrence (1). Currently, there are many definitions of stricture recurrence after urethroplasty, mainly including changes in urinary flow rate and the ability to pass 16Fr/18Fr diameter cystoscope, etc (2). However, due to the limitations of many conditions, most literature still makes the judgment based on the patient's need for further treatment. According to the literature review results, the stricture recurrence rate is about 6%-28% nowadays with different techniques or materials (3). It is important to note that once the stricture recurred, the success rate of the second operation was significantly reduced. In order to find out the possible causes of postoperative stricture recurrence, and some prognostic factors such as BMI, length of stricture, previous urethroplasty history, direct visual internal urethrotomy (DVIU) history have been reported (4). However, in addition to these risk factors that have a strong role in promoting stricture recurrence, there are still some risk factors that are relatively mild or need a long time for stricture promotion, which can only be described by a large sample size of clinical research.

Many studies have pointed out that the use of tobacco, whether it is the inhalation of cigarettes or the use of tobacco powder or e-cigarettes, will increase the level of inflammation in the body. The increase in the level of inflammation in the body is closely related to the formation and aggravation of scars, The increase in the level of inflammation in the body is closely related to the formation and exacerbation of scars, which may further increase the possibility of recurrence of stricture after urethroplasty (5). In 2010, a study pointed out tobacco consumption may lead to stricture recurrence after urethroplasty. However, in many retrospective studies, whether in univariate analysis or multivariate analysis, the role of smoking in the stricture recurrence after the urethroplasty has not been uniformly described. Some studies even mentioned that smoking could be helpful for scar healing (6). Therefore, the objective of this paper was to conduct a meta-analysis based on the reported data to obtain a regular assessment of the relationship between smoking and stricture recurrence after the urethroplasty.

It is worth noting that the statistical analysis of many retrospective studies of risk factors is usually divided into two parts, namely univariable factor regression analysis and subsequent multivariable regression analysis. Suppose only meta-analysis is performed on the results of multivariate regression analysis. In that case, it may lead to obvious selection bias (many studies only include variables that are significant in univariate regression in multivariate regression). Therefore, the results of single factor regression and multivariate regression analysis were combined separately in this study to get a more comprehensive result.

## MATERIALS AND METHODS

The literature collection, data extraction, merging, and subgroup analysis methods used in this study are similar to those of our previous published studies (7).

### Literature search and inclusion criteria

This meta-analysis was performed according to the principle of preferred reporting items for systematic reviews and meta-analysis (PRISMA) (8). This meta-analysis has been registered at PROSPERO (registration number: CRD42021277661). We conducted a pre-analysis to assess feasibility before entirely conducting this meta-analysis. When a preliminary literature search is carried out, and it is clear that a considerable number of high-quality, relevant studies have been published, we will conduct a follow-up detailed search and data sorting and analysis. That is why in PROSPERO registration, we truthfully mentioned that formal screening of search results against eligibility criteria and risk of bias (quality) assessment began before submission to PROSPERO. Pubmed, Embase, Web of Science, and Cochrane Library were searched to identify potential studies. The latest search date was July 1, 2022. Keywords included urethroplasty, smoking, smoker, tobacco consumption, and stricture recurrence. Furthermore, the reference part of every candidate literature was manually screened to find possible data sources.

Detailed inclusion criteria were as follows: Patients were treated with onlay with buccal mucosa or penile fasciocutaneous flap, oral mucosa, or any other type of substitution urethroplasty anastomotic urethroplasty or any combined urethroplasty techniques for anterior or posterior urethral strictures. Odds ratio (OR), hazard ratio (HR), and relative risk (RR) with a 95% confidence interval (CI) of risk factors should be offered or could be calculated. Only English-written studies were included. Exclusion criteria: Reviews, meta-analyses, letters, comments, case serials, and conference abstracts were excluded. Studies focused on hypospadias and pediatric patients and published earlier than 2000 were excluded. Studies that did not contain regression information or enough data to be used for secondary analysis were excluded. Since few studies offered detailed smoking history information such as tobacco type (cigarettes or electronic cigarettes, etc.), smoking time, or whether current smoking, was smoking was not explicitly defined in this study. Because many studies based on adult cases did not strictly distinguish the cause of urethral stricture, this study did not exclude articles according to the cause of urethral stricture. The study was included in the analysis when the original study's smoking history variable was present. Two independent authors carried out all the title screening, abstract screening, and full-text review.

### Research Quality Evaluation

All included studies were evaluated by Newcastle-Ottawa Scale (NOS) system, and two independent reviewers performed the evaluation procedure. Disagreements between the two authors should be determined by the third author (TJ)(9). According to the NOS, 7-9 score studies were considered high-level quality, 5-6 score studies were considered moderate-level, and <5 score studies were low-level quality. Low-level quality studies should not be involved in the meta-analysis.

### Meta-analysis

Based on univariate and multivariate analysis results in this study, the relationship between smoking and stricture recurrence was pooled in a meta-analysis. All analysis was powered by Stata 15.0 software (Stata Corporation, College Station, TX, U.S.A.). Statistical significance was defined as P<0.05 in this study. Pooled estimates larger than 1 indicated that smoking would make patients more vulnerable to stricture recurrence. Heterogeneity was evaluated by I^2^. When I^2^ was larger than 50%, heterogeneity could be significant. If significant heterogeneity was detected, a random effect model should be applied. The primary outcomes were displayed with a forest plot. Subgroup analysis was also performed to get more detailed information. The variables included in subgroup analysis mainly include the research area, the number of participants, the recurrence rate of stricture, the type of estimates, the location of the stricture, and the type of urethroplasty surgery.

Furthermore, sensitivity analysis was performed to test the stability of meta-analysis results, and publication bias was tested by Egger's and Begg's tests. Funnel plots were used for publication-bias visual identification. After data synthesis, the final effect size should be the relative risk. This is a meta-analysis that tried to combine regression estimates. In many retrospective studies, only significant factors in the univariate logistic or Cox regression would be included in the multivariate logistic or Cox regression. Combining multivariate analysis results will undoubtedly bring selection bias if we only combine multivariate analysis results. It is necessary to perform a meta-analysis based on univariate analysis results.

The previously published preprint has detailed and described all the analysis procedures (Research Square, 10.21203/rs.2.23580/v1).

## RESULT

### Study selection

**Figure 1:**
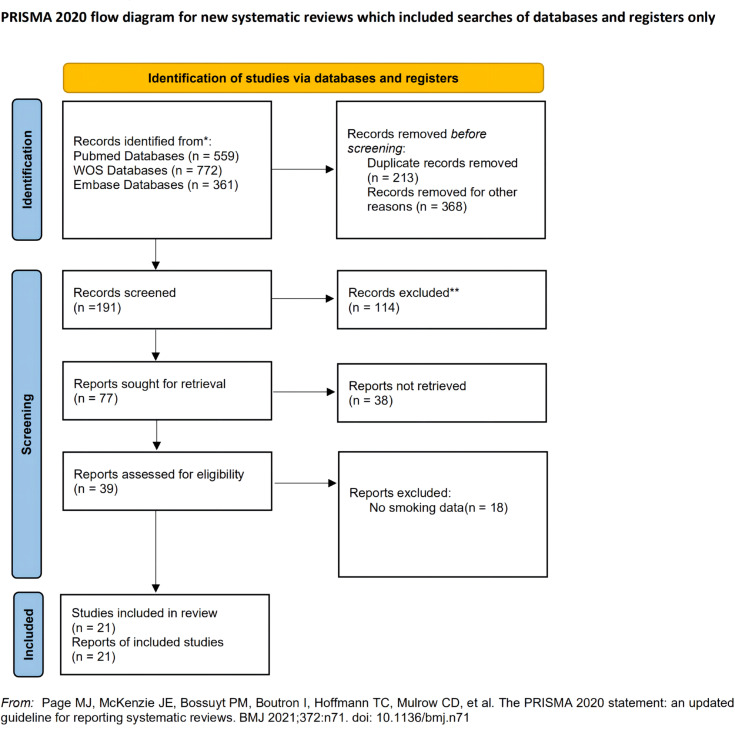
Study searching flow chart.

One thousand three hundred twenty-nine studies were identified from databases in total. 14 of included studies were carried out in the North American region, and 7 were carried out in other regions. Thirteen studies were carried out in recent 5 years, and no study earlier than 2000. Four studies focused on the anterior urethra, 5 on the bulbar urethra, 2 on the posterior urethra, and 7 studies did not limit stricture locations. Almost all studies did not define the cause of stricture. 8 of included studies used techniques for substitution urethroplasty with different materials, 3 of included studies focused on anastomotic urethroplasty. After duplicate removal, abstract screening, and full-text reading, 21 studies were finally involved in this meta-analysis. The detailed screening procedure is displayed in Figure-1. There were 18 studies (total 5811 patients) contained smoking-stricture recurrence univariate analysis information(3, 10-26), 11 studies (total 3167 patients) contained multivariable analysis information(10, 12, 15, 16, 18-20, 27-30). Out of 20 involved studies, 19 studies are retrospective cohort studies, and only 1 study was prospectively designed. Detailed baseline information and research quality evaluation are shown in Table-1 and Table-S1 separately.

For more information, visit: http://www.prisma-statement.org/

**Table 1:** Characteristics of studies included in the meta-analysis.

Author	Year	Country	Stricture location	Study design	Techniques applied	Median/Mean follow (months)	Sample size	Recurrence number	Mean or median age (year)	Definition of stricture recurrence	NOS score
Verla, et al. (25)	**2020**	**Belgium**	Anterior urethral stricture	**PCS**	Anastomotic urethroplasty/ Buccal mucosa graft/ Fasciocutaneous flap/combined technique	**62**	**474**	**81**	**NR**	**symptoms or an** **obstructive voiding curve on urofowmetry (<15 mL/s)**	**7**
Kinnaird, et al. (27)	2014	Canada	Anterior/posterior urethral stricture	RCS	NR	52	604	56	44.5	Cystoscopic evaluation	5
Breyer, et al. (12)	2009	USA	Anterior/posterior urethral stricture/combined stricture	RCS	Anastomotic urethroplasty/ Buccal mucosa graft/ Fasciocutaneous flap/combined techniques	70	381	60	41.5	peak urinary flow less than 15 cc per second and/or the radiographic evidence of stricture and further need for urethral instrumentation	8
Viers, et al. (13)	2017	USA	bulbar urethral stricture	RCS	Excision + primary anastomosis/Substitution	64	514	74	49	the need for recurrent urethral interventions such as endoscopic treatment, subsequent catheterization or repeat urethroplasty	5
Christopher G. Keith (16)	2019	USA	bulbomembranous urethra	RCS	Primary anastomosis	30.7	116	22	72.3	Recurrence was defined by recurrent stricture ≤ 16F in caliber on cystoscopy, stricture on VCUG, and/or operative intervention for urethral stricture disease	7
Chapman, et al. (17)	2017	Canada	bulbar stricture	RCS	BMG Onlay/ Flap Onlay/ Augmented Anastomosis/ Anastomotic/ Combined Tissue plasty	65.4	596	40	44.3	cystoscopic evaluation (inability to easily pass a 16Fr cystoscope)	7
Whitson, et al. (18)	2007	USA	Anterior Urethral Stricture	RCS	fasciocutaneous flap urethroplasty	87.6	124	26	48	Subjective and objective improvement in urinary flow, absence of radiographic evidence of stricture, and no further need for urethral instrumentation.	5
Liu, et al. (30)	2015	USA	Fossa navicularis/ Penile/ Bulbomembranous/ Panurethral strictures	RCS	Dorsal onlay/ Ventral onlay/ Staged urethroplasty	59.3	239	66	42.9	A stricture <16F in caliber was visually present in the reconstructed segment of urethra on cystoscopy	8
Han, et al. (19)	2015	USA	Posterior urethral stricture	RCS	Excision/primary anastomosis/ Dorsal onlay (including augmented anastomotic)/ Ventral onlay/ Staged/ Combined/flap/miscellaneous	62	237	60	42.9	patient-reported recurrent urinary symptoms and urethral caliber less than 18-Fr on cystoscopy, and/or need for any subsequent intervention (including dilation, endoscopic urethrotomy or repeat urethroplasty)	5
Kahokehr, et al. (11)	2018	USA	bulbar urethral stricture	RCS	Excision + primary anastomosis/ Augmented anastomotic repair/ Onlay	28	395	23	43.41	Stricture recurrence was defined as the need for further intervention in the postoperative period as diagnosed with cystoscopy and/or RUG	5
Levy, et al. (29)	2017	USA	Bulbar/ Meatus/Fossa/ Membranous/ Penile stricture	RCS	Excision + primary anastomosis/BMG dorsal onlay	21.6	322	22	44.2	the freedom from additional procedures after urethroplasty	5
Mathur, et al. (24)	2014	India	Anterior (penile+ bulbar)/ Posterior (membranous/bulbomembranous)/ Panurethra strictures	RCS + prospective data	single-stage penile preputial flap urethroplasty	42	58	11	42.2	Patient reported symptoms and retrograde urethrography	5
Sinha, et al. (23)	2010	India	Penile/Bulbar/ Bulbopenile/ Pananterior stricture	NRPCS	Oral mucosa graft Urethroplasty	18.2	42	11	40.2	failure was defined as the need to carry out any intervention or invasive procedure in the urethra following the complaint of decreased urinary flow by the patient	5
Manjunath, et al. (10)	2019	USA	Fossa navicularis/ Penile urethra/ Bulbar urethra/ Membranous urethra stricture	RCS	Excision and primary Anastomosis, substitution urethroplasty performed utilizing buccal mucosa, tunica vaginalis, or abdominal wall skin grafts	52.5	398	78	42.8	patient-reported recurrent urinary symptoms and urethral caliber less than 16-Fr on cystoscopy, and/or need for any subsequent intervention (including dilation, endoscopic urethrotomy, or repeat urethroplasty)	7
Figler, et al. (14)	2013	USA	Bulbar urethra stricture	RCS	Urethroplasties With Buccal Mucosa Graft	35.7	103	19	40.8	the need for endoscopic or open revision of the reconstruction or the placement of a suprapubic catheter for urinary retention	5
Meyer, et al. (15)	2020	Germany	Anterior urethra stricture	RCS	One-stage Buccal Mucosal Graft Urethroplasty	32	517	76	53.7	need for any intervention	5
Redmond, et al. (3)	2020	Canada	Bulbar Urethral Strictures	RCS	Dorsal onlay repair, anastomotic urethroplasty with dorsal BMG	78.9	507	31	45.4	Inability to easily pass a 16Fr flexible cystoscope.	7
Karapanos, et al. (20)	2021	Germany	Anterior urethra stricture	RCS	tissue-engineered oral mucosa graft urethroplasty	7	77	24	60	the need for any further treatment for recurrent stricture or a Qmax <15 mL/s	5
Davenport, et al. (21)	2019	USA	Bulbar urethral strictures	RCS	Excision and primary anastomosis	52.4	853	85	53.1	Functional emptying and lack of need for further endoscopic or open re-operative management	5
Shalkamy, et al. (22)	2020	Egypt	Anterior urethral stricture	RCS	BMG urethroplasty	49.77	266	34	37.71	The need for further intervention, including urethral dilation, was considered stricture recurrence.	5
Ma, et al. (29)	2021	China	Posterior urethra stricture	RCS	Excision and primary anastomosis	49	153	31	45	Patients received further surgical intervention or instrumentation, such as urethra dilation, urethrotomy, or any endoscopic management, patients who had any medical record about postoperative endoscopy confirmed urethral stricture, patients reported failure.	5

**RCS** = Retrospective Cohort Study; **NRPCS** = Non-Randomized Prospective Cohort Study; **NR** = Not Reported; **NOS** = Newcastle-Ottawa Scale; **BMG** = buccal mucosa graft.

### Meta-analysis based on univariable analysis

**Figure 2:**
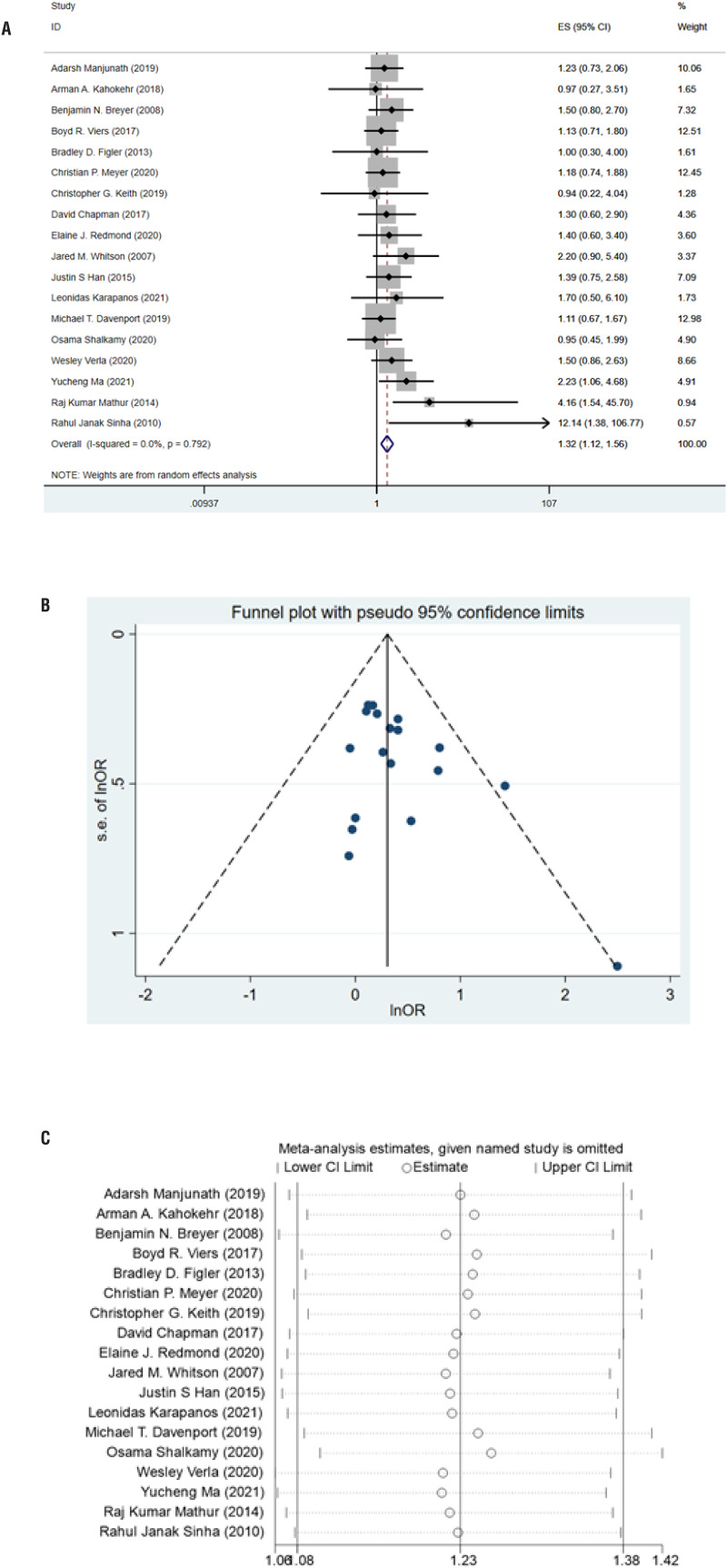
A) Forest plot of crude estimate meta-analysis between smoking and stricture recurrence. B) Funnel plot of crude estimate meta-analysis. C) Sensitivity analysis of crude estimate meta-analysis.

In terms of univariate analysis, 18 studies containing 5811 patients exploring the association between smoking and stricture recurrence after urethroplasty. According to the overall meta-analysis result, smoking can make patients more vulnerable to stricture recurrence (RR=1.32, 95%CI: 1.12-1.56, P=0.001) with no significant heterogeneity found (I^2^=0.0%, p=0.792) (Figure-2A). No significant publication bias was found according to Egger's test (P=0.058) and Begg's test (P=0.112) and was shown in the funnel plot (Figure-2B). Sensitivity analysis showed that the results were not significantly changed by omitting included studies one by one (Figure-2C). Further subgroup analysis results were displayed in Table 2. We found that only when strictures were located in the anterior urethra smoking could significantly negatively affect stricture recurrence (RR=1.42, 95%CI:1.03-1.96, P=0.033).

**Table 2:** Subgroup analyses of meta-analysis.

Pooled results based on univariable analysis						Pooled results based on multivariable analysis			
		Pooled estimate for stricture recurrence		Heterogeneity		Pooled estimate for stricture recurrence		Heterogeneity	
**Subgroup**		OR (95%CI)	P value	I2	P value	OR (95%CI)	P value	I2	P value
**Region**									
	North American	1.26 (1.03, 1.54)	0.026	0.0%	0.983	1.29 (0.91, 1.84)	0.154	38.0%	0.126
	Other	1.55 (1.07, 2.24)	0.020	29.5%	0.203	1.64 (0.78, 3.44)	0.192	56.5%	0.100
**Patient number**									
	>300	1.24 (1.02, 1.50)	0.028	0.0%	0.993	1.12 (0.86, 1.47)	0.400	0.0%	0.479
	≤ 300	1.61 (1.14, 2.28)	0.007	10.4%	0.349	1.84 (1.04, 3.26)	0.036	51.0%	0.070
**Recurrence rate**									
	>10%	1.37 (1.14, 1.65)	0.001	0.0%	0.596	1.44 (1.01, 2.05)	0.042	48.1%	0.052
	≤10%	1.18 (0.83, 1.66)	0.359	0.0%	0.947	1.05 (0.59, 1.90)	0.862	0.0%	0.748
**Effect type**									
	HR	1.33 (1.10, 1.61)	0.004	0.0%	0.875	1.61 (1.02, 2.56)	0.043	59.2%	0.031
	OR	1.37 (0.94, 1.99)	0.102	13.0%	0.330	1.08 (0.74, 1.56)	0.702	0.0%	0.649
**Stricture Location**									
	Anterior	1.42 (1.03, 1.96)	0.033	0.0%	0.650	2.45 (0.70, 8.66)	0.163	76.9%	0.013
	Bulbar	1.16 (0.83, 1.63)	0.375	0.0%	0.981	/	/	/	/
	Posterior	1.83 (0.90, 3.73)	0.094	6.9%	0.300	2.26 (1.13, 4.52)	0.021	0.0%	0.887
	Not specified	1.32 (1.00, 1.76)	0.054	16.7%	0.303	1.12 (0.85, 1.47)	0.428	0.0%	0.612
**Urethroplasty type**									
	Substitution	1.44 (1.01, 2.05)	0.046	15.2%	0.310	1.70 (0.80, 3.65)	0.170	68.3%	0.024
	Without substitution	1.36 (0.84, 2.22)	0.216	25.3%	0.262	2.26 (1.13, 4.51)	0.021	0.0%	0.887
	Not specified	1.30 (1.03, 1.63)	0.025	0.0%	0.981	1.15 (0.85, 1.55)	0.365	0.0%	0.497
**Recurrence definition**									
	Multiple definitions	1.47 (1.14, 1.89)	0.003	0.0%	0.685	1.81 (1.00, 3.27)	0.051	58.4%	0.035
	Single definition	1.23 (0.99, 1.53)	0.066	0.0%	0.719	1.18 (0.86, 1.61)	0.311	0.0%	0.398

### Meta-analysis based on multivariate analysis

Based on multivariate analysis, the association between smoking and stricture recurrence after urethroplasty was explored in 11 studies containing 3167 patients. According to the overall meta-analysis result, smoking can make patients more vulnerable to stricture recurrence (RR=1.35, 95%CI: 1.002-1.81, P=0.049) with no significant heterogeneity found (I^2^=37.4%, p=0.09) (Figure-2A). No significant publication bias was found according to Egger's test (P=0.087) and Begg's test (P=0.062) and was shown in the funnel plot (Figure-2B). Sensitivity analysis indicated that this data synthesis might not be exactly stable (Figure-2C). Further subgroup analysis results were displayed in Table-2. Interestingly, smoking was no longer significant after anterior urethra stricture treatment (RR=2.45, 95%CI:0.70-8.66, P=0.163) but significant after posterior urethra treatment (RR=2.26, 95%CI:1.13-4.52, P=0.021) when the data were pooled using the results of the multivariable regression.

## DISCUSSION

Urethral stricture is a kind of pathological stricture of the urethra, which can limit fluid transportation. Since the male urethra is significantly longer than the female urethra, and the posterior urethra is hidden in the pelvis, the urethral stricture can always bring many troubles to patients and urologists. Urethral stricture is a common urinary disease for males. There are 229-627 cases in every 100000 people, and in some susceptible groups, such as elderly men, the prevalence rate is as high as 0.6% (31). As one of the main methods to treat urethral stricture, there are many ways to implement urethroplasty, including primary anastomosis and substitution implantation. However, although many different surgical methods have been developed for different stricture degrees, lengths and locations, the success rate is still only 72% - 94%. Therefore, it is very important to find out the risk factors of recurrence of urethral stricture after urethroplasty and to prevent them. Some risk factors such as the length of stricture and etiology have attracted the attention of urologists, but other factors such as tobacco consumption have not been evaluated carefully

This meta-analysis revealed that tobacco consumption could increase the chance of stricture recurrence based on univariate and multivariate analyses. In the multivariate analysis stage, the sensitivity analysis result was not exactly stable, indicating that more multivariate analysis studies and adjusted estimates between smoking and stricture recurrence were required. In the univariate subgroup analysis, we found that anterior urethral stricture is most likely to be affected by smoking, increasing the risk of recurrence of the stricture. Similarly, patients who use substitution urethroplasty are more likely to be affected by smoking. However, similar effects were not found in the subgroup analysis based on the results of multivariable regression. This may be since fewer original studies provide the results of multivariate regression analysis. It was worth noting that in the subgroup analysis, we found that the pooled results based on HR always showed statistical significance, which may mean that the influence of smoking on the recurrence might have time-dependent distribution (For example, it was easier to follow the law for a long time after surgery). However, since the primary studies did not provide a KM curve, we could not perform further analysis.[Fig f3]

**Figure 3 f3:**
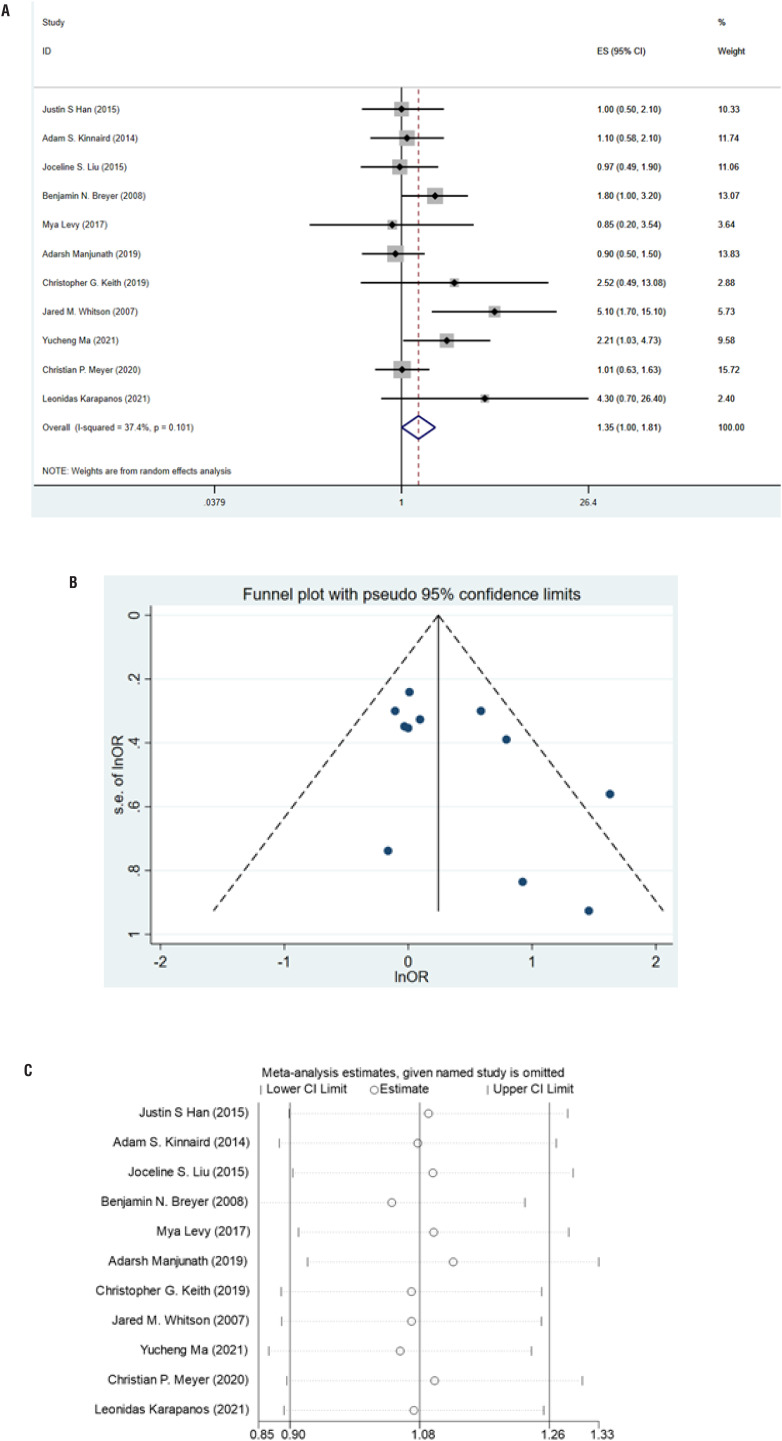
A) Forest plot of adjusted estimate meta-analysis between smoking and stricture recurrence. B) Funnel plot of adjusted estimate meta-analysis. C) Sensitivity analysis of adjusted estimate meta-analysis.

Many studies have pointed out that smoking has a negative effect on healing wounds that cannot be ignored. Smoking temporarily reduces tissue oxygenation and aerobic metabolism. The inflammatory healing response is weakened by reducing inflammatory cell chemotaxis, migration function, and oxidative sterilization mechanism. In addition, the release of proteolytic enzymes and inhibitors would be unbalanced when the tissue was hypoxic or inflammatory substances were present. In addition to down-regulating collagen synthesis and deposition, reduced fibroblast migration and proliferation can also impair the proliferative response. A wound that delays healing would inevitably lead to repeated chronic local inflammation and tissue remodeling, which may be an important reason for stricture recurrence.

Furthermore, for patients who receive oral mucosa graft Urethroplasty (OMGU), a smoking history will make the general state of oral mucosa worse, leading to poor graft survival after OMGU operation and ultimately leading to an increase in stricture recurrence rate. In urethroplasty using oral mucosa as a substitution, the effect of tobacco on the oral mucosa must also be considered. Although, in some studies, patients have been advised to avoid smoking before taking oral mucosal materials, long-term smoking history can hardly avoid the impact on the viability of oral mucosa, which may further increase the impact of smoking on oral mucosa and the recurrence of stricture.

In some current urological guidelines, the effect of smoking on the stricture recurrence after urethroplasty is not mentioned. Although EAU related narratives had clearly mentioned that smoking had an important influence on the choice of surgery, in the follow-up part, it was still not mentioned that smoking is an important risk factor for postoperative recurrence.

According to the results of this meta-analysis, urologists should guide urethroplasty patients to quit smoking before and after the operation to improve the overall success rate. Some potential limitations of this study should be presented. First, although some prospective data was involved, all the included studies are retrospective. Second, although it was recognized in statistical methodology, it is still possible to bring some additional bias by combining HR and OR to get RR estimates. Third, since smoking can directly damage oral mucosa, OMGU patients with a smoking history may have a higher recurrence ratio. It is also worth noting that the many other factors, such as postoperative complications and other factors, obviously play an important role in the recurrence of strictures, but not every primary study has fully adjusted the effects of these factors on smoking. However, in this meta-analysis, since many studies didn't offer detailed information about the OMGU technique, so OMGU subgroup analysis was not performed, further high-level evidence about smoking's effect on OMGU is needed.

## CONCLUSION

Our study shows that smoking can increase stricture recurrence risk after the urethroplasty. Quitting smoking may be a good option for patients undergoing urethroplasty surgery.

## ABBREVIATIONS

NOS: = Newcastle-Ottawa Scale
HR: = Hazard Ratio
OR: = Odds Ratio
RR: = Relative Risk
CI: = Confidence Interval
DVIU: = Direct Visual Internal Urethrotomy
PRISMA: = Preferred Reporting Items for Systematic Reviews and Meta-analysis
OMGU: = Oral Mucosa Graft Urethroplasty
RCS: = Retrospective Cohort Study
NRPCS: = Non-Randomized Prospective Cohort Study
B.M.G.: = Buccal Mucosa Graft

## Appendix

**Table S1:** Newcastle-Ottawa scale score of the reviewed studies.

Study	Selection (4 stars)				Comparability (2 stars)	Outcome (3 stars)			Total score
	Representativeness score of the stricture recurrence	Selection of the stricture recurrence	Ascertainment of stricture recurrence	Demonstration that outcome of interest was not present at start of study	Comparability of cohorts based on the design or analysis	Assessment of outcome	Was follow up long enough for outcomes to occur?	Adequacy of follow up of cohort	
Verla, et al. (25)	/	★	★	★	★	★	★	★	7
Kinnaird, et al. (27)	/	★	/	★	★	/	★	★	5
Breyer, et al. (12)	★	★	★	★	★	★	★	★	8
Viers, et al. (13)	/	★	/	★	★	/	★	★	5
Keith, et al. (16)	/	★	★	★	★	★	★	★	7
Chapman, et al. (17)	/	★	★	★	★	★	★	★	7
Whitson, et al. (18)	/	★	/	★	★	/	★	★	5
Liu, et al. (31)	★	★	★	★	★	★	★	★	8
Han, et al. (19)	/	★	/	★	★	/	★	★	5
Kahokehr, et al. (11)	/	★	/	★	★	/	★	★	5
Levy, et al. (29)	★	★	/	★	★	/	/	★	5
Mathur, et al. (24)	★	★	/	★	/	/	★	★	5
Sinha, et al. (23)	★	★	/	★	★	/	/	★	5
Manjunath, et al. (10)	/	★	★	★	★	★	★	★	7
Figler, et al. (14)	★	★	/	★	★	/	/	★	5
Meyer, et al. (15)	★	★	/	★	/	/	★	★	5
Redmond, et al. (3)	/	★	★	★	★	★	★	★	7
Karapanos, et al. (20)	/	★	/	★	★	/	★	★	5
Davenport, et al. (21)	/	★	/	★	★	/	★	★	5
Shalkamy, et al. (22)	★	★	/	★	★	/	/	★	5
Ma, et al. (29)	★	★	/	★	★	/	/	★	5
